# Polycythemia in a Patient With Atonic Bladder and Hydronephrosis

**DOI:** 10.7759/cureus.18094

**Published:** 2021-09-19

**Authors:** Vatsala Katiyar, Talha Aijaz, Prasanth Lingamaneni, Ishaan Vohra, Kamila Cisak

**Affiliations:** 1 Division of Medical Oncology and Hematology, Department of Internal Medicine, James Graham Brown Cancer Center, University of Louisville School of Medicine, Louisville, USA; 2 Department of Internal Medicine, John H. Stroger, Jr. Hospital of Cook County, Chicago, USA; 3 Division of Gastroenterology, Department of Internal Medicine, University of Illinois College of Medicine at Peoria, Peoria, USA

**Keywords:** polycythemia, hypoxia inducible factor, erythropoietin, hydronephrosis, secondary erythrocytosis, secondary polycythemia

## Abstract

Kidneys influence the production of red blood cells by secreting most of the erythropoietin (EPO) in adults. Consequently, renal diseases often impact erythropoiesis and hemoglobin levels. Chronic kidney diseases lead to anemia due to EPO deficiency. However, erythrocytosis can occur in patients with cystic diseases of the kidney and renal artery stenosis due to upregulation of hypoxia-inducible factors (HIFs) and increased EPO production. Here, we present a patient with secondary polycythemia who was found to have atonic bladder and hydronephrosis. Resolution of hydronephrosis led to the reversal of erythrocytosis, highlighting the intricate regulation of red cell production.

## Introduction

Polycythemia is a state of abnormally elevated red blood cell mass defined as hemoglobin >16.5 g/dL in men and >16.0 g/dL in women or a hematocrit >49% in men and >48% in women [1]. Secondary erythrocytosis occurs in instances of tissue hypoxia or inappropriate autonomous erythropoietin (EPO) secretion by tumors. Renal diseases also lead to polycythemia by upregulating EPO production when the oxygen sensing mechanism is impaired [2]. Here, we report a patient with hydronephrosis and polycythemia whose hemoglobin normalized after resolution of hydronephrosis.

## Case presentation

A 60-year-old male was referred to hematology clinic for erythrocytosis. A month before the clinic appointment, he experienced a motor vehicle accident (MVA) and went to the emergency room (ER), where elevated hemoglobin was first detected. He had not seen a physician in many years prior to the MVA.

**Figure 1:**
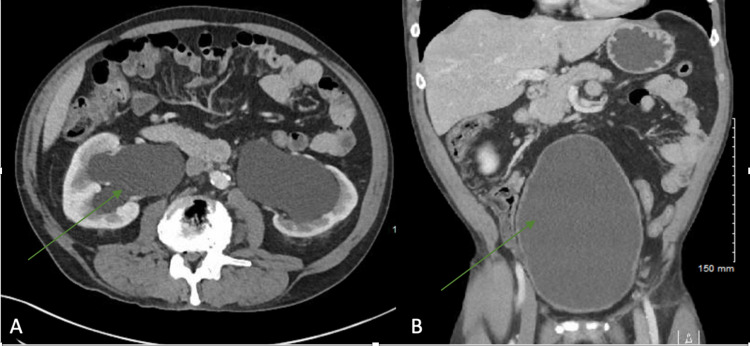
Computed tomography scan of abdomen of (A) axial and (B) coronal sections showing hydroureteronephrosis and distended bladder.

**Figure 2:**
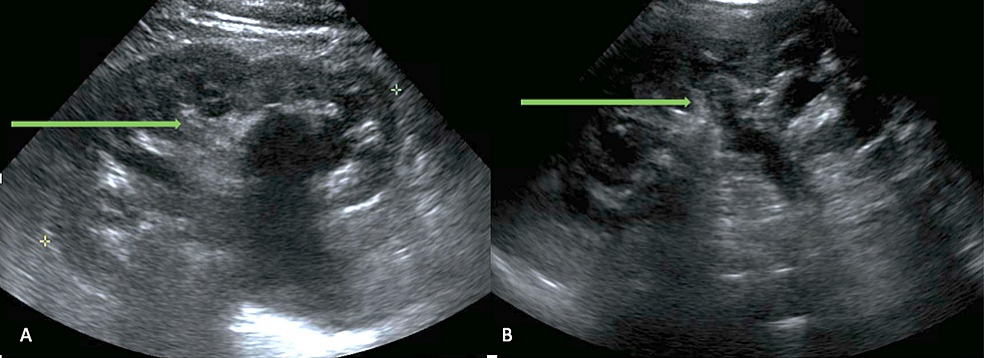
Ultrasound (sagittal view) of left kidney (A) and right kidney (B) showing improvement in hydronephrosis.

Laboratory values during the ER visit are reported in Table 1. There was no evidence of acute traumatic injury on whole-body computed tomography, but it showed a markedly distended urinary bladder with severe bilateral hydronephrosis concerning outflow obstruction (Figures 1A, 1B). He was discharged with a foley catheter and urology follow-up. On a repeat kidney ultrasound, bilateral hydronephrosis had decreased (Figures 2A, 2B). Prostate-specific antigen (PSA) was normal, and cystoscopy did not reveal any evidence of malignancy. He was diagnosed with idiopathic atonic bladder and was advised to continue intermittent catheterization. The patient continued to have persistent erythrocytosis with subsequent hemoglobin being 20.4 g/dL and 19.2 g/dL.

**Table 1:** Pertinent laboratory values.

Lab parameters	Values in ER	Values in hematology clinic	Reference range
Hemoglobin	20.7	18.7	7-18 g/dL
Hematocrit	59.1	54.1	40-51%
White blood cells	6.8	6.5	4.1-10.8 k/uL
Platelets	200	219	140-370 k/uL
Creatinine	1.61	1.40	0.64-1.27 mg/dL
Lactate dehydrogenase	-	169	100-242 U/L
Erythropoietin	-	3.3	2.6-18.5 mIU/mL

During his hematology clinic visit, he only reported lower urinary tract symptoms and required intermittent catheterization. He denied dizziness, headaches, pruritis, erythromelalgia, night sweats, fevers, vomiting, diarrhea, weight loss, and previous blood clots. He did not use any over-the-counter medications or anabolic steroids. There was no family history of blood disorders. He had a 40-pack year smoking history and reported chronic alcohol use. His body mass index was 23.6 kg/m^2^, and there were no symptoms suggestive of obstructive sleep apnea. Laboratory workup during the hematology visit is detailed in Table 1. Peripheral blood smear showed erythrocytosis with no abnormal cell morphology. Since the EPO level was at the lower end of normal, JAK2 V617F, JAK2 exon 12-14, CALR, MPL mutations were sent but not detected.

**Figure 3:**
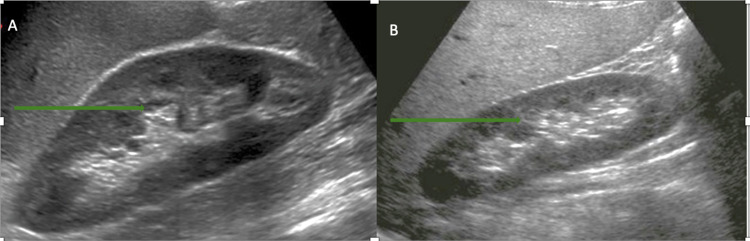
Ultrasound (sagittal view) of left kidney (A) and right kidney (B) demonstrating resolution of hydronephrosis.

He was advised to quit smoking, but unfortunately, he continued to smoke. At a subsequent two-month follow-up, his hemoglobin had down trended to 14.7 g/dL, and at six-month follow-up, it was 15.8 g/dL. There was no evidence of bleeding. Other cell lines continued to stay within normal limits. Repeat kidney ultrasound showed normalization of kidney size (Figure 3A, 3B). He was deemed to have polycythemia secondary to local renal ischemia induced by renal tubular compression in the setting of hydronephrosis.

## Discussion

Hypoxia-driven erythrocytosis is associated with increased EPO production as opposed to polycythemia vera, where EPO levels are characteristically low [2]. EPO is a glycoprotein hormone composed of a single 165 amino acid residue chain linked to four glycans and is primarily secreted by the peritubular fibroblasts in the renal cortex [3]. EPO gene expression is tightly regulated by a complex mechanism involving hypoxia-inducible factors (HIF). As the blood oxygen concentration declines, the ubiquitination and proteasomal degradation of HIF-1 decreases, thereby raising its levels. It binds to a cis-acting hypoxia response element present at the 3" flanking region of the human EPO gene. In turn, the elevation in HIF-1 leads to increased transcription of the EPO gene and a rise in its levels. Erythropoietin binds to its receptor on the erythroid progenitor cells, which initiates the signal transduction cascade and ultimately stimulates the survival, proliferation, and differentiation of erythroid precursors [4,5].

Increased HIF and consequent high EPO levels can occur in instances of true hypoxia from chronic pulmonary disease, right to left cardiac shunts, sleep apnea and chronic carbon monoxide poisoning, or compromised oxygen detection due to diseases intrinsic to the kidney such as renal artery stenosis, cysts and hydronephrosis [2]. In addition, inappropriate autonomous EPO production by tumors like hepatocellular carcinoma, renal cell carcinoma, and pheochromocytoma can also lead to secondary erythrocytosis [2].

Our patient had erythrocytosis as well as severe hydroureteronephrosis and atonic bladder on presentation. He started intermittent self-catheterization, which led to the resolution of hydronephrosis and a decline in hemoglobin levels. The proposed mechanism for polycythemia in hydronephrosis is intrarenal hypoxia caused by tubular compression, which is reversed with the withdrawal of the stimulus [6]. Interestingly, in a patient with leukemic infiltration of the kidney and secondary polycythemia, increased HIF 1a expression has been demonstrated in kidney cells, suggesting that renal tubules become ischemic when compressed and stimulate EPO synthesis [7].

In addition to kidney disease, our patient had a significant smoking history, which is known to cause erythrocytosis through multiple mechanisms. Smoking not only leads to chronic lung disease and increased carbon monoxide levels causing absolute polycythemia, but it also triggers volume contraction giving rise to relative polycythemia [8]. However, our patient did not have any history or imaging findings suggestive of lung disease, and his hemoglobin levels decreased despite persistent smoking, prompting us to look for other etiologies.

As EPO level was at the lower end of normal, there was concern about polycythemia vera. Hence, JAK2 V617F and JAK2 exon 12-14 mutations testing was done. Bone marrow biopsy was deferred as he did not have detectable mutations and hematocrit normalized. Consequently, we concluded that his erythrocytosis was in fact, related to hydronephrosis.

## Conclusions

Even though hydronephrosis is a common diagnosis, its association with secondary erythrocytosis is not usually seen in clinical practice. The amount of residual non-ischemic renal tissue could play a role in the infrequent occurrence of the two conditions together. Moreover, other pathways apart from tubular compression and HIF upregulation could contribute to polycythemia in patients with hydronephrosis and need further investigation.
